# Carbon sandwich preparation preserves quality of two-dimensional crystals for cryo-electron microscopy

**DOI:** 10.1093/jmicro/dft038

**Published:** 2013-07-23

**Authors:** Fan Yang, Kazuhiro Abe, Kazutoshi Tani, Yoshinori Fujiyoshi

**Affiliations:** 1 Faculty of Science, Department of Biophysics, Kyoto University, Oiwake, Kitashirakawa, Sakyo-ku, Kyoto 606-0852, Japan; 2 Cellular and Structural Physiology Institute, Nagoya University, Chikusa-ku, Nagoya 464-8601, Japan; 3 Department of Medicinal Science, Graduate School of Pharmaceutical Science, Nagoya University, Chikusa-ku, Nagoya 464-8601, Japan

**Keywords:** cryo-electron microscopy, electron crystallography, H^+^,K^+^-ATPase, two-dimensional crystals, membrane proteins

## Abstract

Electron crystallography is an important method for determining the structure of membrane proteins. In this paper, we show the impact of a carbon sandwich preparation on the preservation of crystalline sample quality, using characteristic examples of two-dimensional (2D) crystals from gastric H^+^,K^+^-ATPase and their analyzed images. Compared with the ordinary single carbon support film preparation, the carbon sandwich preparation dramatically enhanced the resolution of images from flat sheet 2D crystals. As water evaporation is restricted in the carbon-sandwiched specimen, the improvement could be due to the strong protective effect of the retained water against drastic changes in the environment surrounding the specimen, such as dehydration and increased salt concentrations. This protective effect by the carbon sandwich technique helped to maintain the inherent and therefore best crystal conditions for analysis. Together with its strong compensation effect for the image shift due to beam-induced specimen charging, the carbon sandwich technique is a powerful method for preserving crystals of membrane proteins with larger hydrophilic regions, such as H^+^,K^+^-ATPase, and thus constitutes an efficient and high-quality method for collecting data for the structural analysis of these types of membrane proteins by electron crystallography.

## Introduction

Since the first report of the three-dimensional (3D) structure of the membrane protein bacteriorhodopsin [1], electron crystallography of two-dimensional (2D) crystals has become a valuable approach for determining the structure of membrane proteins [2]. The ability to obtain structural information from 2D-ordered arrays makes this approach particularly useful for studies of membrane proteins in the lipid bilayer. Like X-ray crystallography, electron diffraction at high resolution obtained directly from sufficiently large and well-ordered 2D crystals allows the construction of an atomic model [3,4]. For phasing of the diffraction data, and especially for the structural determination of small or disordered 2D crystals, however, extraction of structural information from electron micrographs is a key requirement [2–6].

In addition to the quality of 2D crystals themselves, several factors in sample preparation and data collection, such as lack of specimen flatness, radiation damage, dehydration and image shift due to beam-induced specimen charging, can contribute to degradation of image quality, and thus limit image resolution and efficient data collection [2,7,8]. Accordingly, numerous efforts have been made to improve and overcome these factors. To maintain 2D crystals in the hydrated state, methods for sugar embedding [1] and vitrified ice embedding [9] have been introduced. Trehalose is a sugar best known in specimen preparation for its use as an embedding medium for 2D crystals [2,10,11]. Owing to its high ability to preserve crystals in a vitrified specimen, trehalose-embedding has been applied to many successful structural analyses of proteins such as bacteriorhodopsin [12] and aquaporins [13–15]. In contrast to these examples, however, 2D crystals of membrane proteins with large hydrophilic portions are much more susceptible to specimen dehydration and also to changes in salt concentrations, and even trehalose-embedding is insufficient to preserve their crystal quality.

The carbon sandwich technique was developed by Koning *et al.* [16] to improve specimen flatness, with some modifications introduced later by Gyobu *et al.* [17]. In the carbon sandwich preparation, a solution containing 2D crystals is placed on a molybdenum grid that is sandwiched between two sheets of symmetric carbon films, and excess liquid is blotted away from the side of the grid with filter paper prior to freezing. It has been demonstrated that this preparation compensates for the image shift that causes beam-induced specimen charging, and therefore dramatically increases the yield of good images obtained at high-tilt angles [17]. Besides its ability to compensate for the image shift, 2D crystals placed between two carbon films are expected to be better preserved in a hydrated state compared with standard single carbon support film preparations [11].

In this paper, using 2D crystals of gastric H^+^,K^+^-ATPase, we demonstrate that the carbon sandwich preparation better maintains the inherent crystal quality in cryo-specimens than a single carbon preparation. Together with its strong compensation effect against image shift due to specimen charging, which is particularly critical when imaging tilted specimens [17], the carbon sandwich preparation technique allows the extraction of high-quality structural information from preserved 2D crystals, thereby enhancing data collection for 3D reconstruction.

## Materials and methods

### Materials

Continuous carbon support films were prepared by depositing carbon on a freshly cleaved mica surface [2] and transferring to molybdenum grids as described by Gyobu *et al.* [17]. Pig gastric H^+^,K^+^-ATPase was purified and used for 2D crystallization as described previously [18].

### Two-dimensional crystallization

Two-dimensional crystals of H^+^,K^+^-ATPase at different states of the transport cycle were produced as described by Abe *et al.* [19,20]. Decylmaltoside (DM)-solubilized H^+^,K^+^-ATPase grown in dialysis buffer containing aluminum fluoride (AlF) produced single-layered sheet crystals, while an octaethyleneglycol dodecylether (C_12_E_8_)-solubilized preparation in berylium fluoride (BeF) with or without the specific inhibitor SCH28080 resulted in thick tubular crystals.

### Specimen preparations

Samples were negatively stained with 2% (w/v) uranyl acetate to screen for crystallization conditions.

The carbon sandwich preparation was performed as described by Gyobu *et al.* [17], with some modifications. A small (∼3 × 3 mm) piece of solid carbon film was floated on dialysis buffer containing 7% (w/v) trehalose and picked up with a molybdenum grid. The side of the grid without the carbon film was carefully wiped using the middle part of a pipette tip to remove excess carbon film from the grid edge. A H^+^,K^+^-ATPase 2D crystal solution (2 μl) was injected on the same side of the grid and mixed on the grid. After removal of excess crystal solution, a second piece of carbon film of ∼2 × 2 mm floated on the same dialysis buffer was picked up with a platinum loop and deposited on the side of the grid without carbon film. Excess liquid was carefully blotted away using pieces of filter paper. The first few pieces of filter paper were placed against the grid edge for more than 20 s to ensure that the liquid was continuously removed from the grid. The blotting step is especially important for optimizing the vitrified ice thickness, which usually takes a total of 5–10 min for highly viscous samples, such as those with glycerol-containing buffer. After removal of excess liquid, the grid was frozen by plunging it into liquid nitrogen. All steps were performed at 4°C.

For single carbon film preparations, a small (∼3 mm^2^) piece of carbon film was floated on dialysis buffer containing 7% (w/v) trehalose and picked up with a molybdenum grid. The solution containing H^+^,K^+^-ATPase 2D crystals (2 μl) was injected on the side of the grid opposite the carbon film and mixed well by pipetting. After removal of excess buffer, the grid was blotted 1 or 2 times with filter paper with each blotting taking 1–5 s, and then dehydrated for 10–30 s depending on the environment, followed by plunge freezing into liquid nitrogen. Optimization of the vitrified ice thickness can be achieved by changing the trehalose concentration, as well as blotting and dehydration times. All steps were performed at 4°C.

### Electron microscopy and image analysis

Negatively stained specimens were imaged using a JEM-1010 transmission electron microscope (JEOL) operated at 100 kV. For cryo-electron microscopy, the images were recorded with a JEM-3000SFF electron microscope (JEOL) equipped with a field emission gun and a super-fluid helium stage [21] and operated at 300 kV. Images were recorded on SO-163 film (Carestream) at a nominal magnification of 40 000× with a 2-s exposure and a total electron dose of 25 electrons Å^−2^. The micrographs were developed for 14 min at 20°C using a full-strength Kodak D19 developer. The quality of the images was assessed by optical diffraction, and selected images were digitized with a SCAI scanner (Zeiss) using a step size of 7 μm. The digitized images were processed with the MRC image-processing programs [22]. The crystal lattices were computationally unbent and corrected for the contrast transfer function (CTF) [6]. Initial CTF parameters for each image were determined by square frequency filtering [23] combined with periodogram averaging [24].

The information of several independent crystals can be extracted from multiple regions in a single film image for the following reasons. Micrographs of tubular or vesicular crystals of H^+^, K^+^-ATPase contained at least two independent crystalline lattices due to their morphology. Each crystalline layer was imperfect, usually containing a certain degree of defects (i.e*.* mosaic crystal). Therefore, the number of micrographs rather than the number of individually processed crystalline lattices is shown in Table 1 to allow consistent and quantitative comparisons of the population of successfully preserved crystals in each sample.

**Table 1.** Statistics of the data collection for structural analysis of H^+^,K^+^-ATPase in different conformations

Conformation	Preparation	Cryo-grids used	Micrographs						Resolution	Phase residual	Ref.
			Tilt angles					Total			
			0°	20°	45°	60°	70°				
*E2*AlF	Single carbon	121	–	–	–	–	–	–	–	–	–

			–	–	–	–	–	–			

			59	10^a^	28^a^	–	–	97			

			328	465	420	–	–	1213			

*E2*AlF	Carbon sandwich	182	7	48	92	118	54	319	6.5 Å	31.5°	[15]

			70	120	202	206	119	717			

			86	205	311	262	217	1081			

			274	584	660	430	349	2297			

*E2*BeF	Carbon sandwich	47	11	20	57	–	–	88	8 Å	33.1°	[24]

			38	24	73			135			

			47	64	90	–	–	201			

			120	156	286	–	–	562			

(SCH)*E2*BeF	Carbon sandwich	97	6	43	128	118	–	295	7 Å	37.1°	[16]

			14	53	203	210		480			

			15	59	278	249	–	601			

			70	90	542	403	–	1105			

(Rb^+^)*E2*AlF	Carbon sandwich	78	3	18	68	57	–	146	8 Å	35.6°	[25]

			9	29	88	116		242			

			42	87	208	202	–	539			

			80	275	410	402	–	1167			

(K^+^)*E2*AlF	Carbon sandwich	125	6	34	54	69	–	163	8 Å	36.9°	[25]

			38	73	118	126		355			

			80	100	180	204	–	564			

			443	588	430	551	–	2012			

Table shows numbers of ‘micrographs’ used for the structural determination of the indicated conformation of H^+^,K^+^-ATPase (see Materials and Methods for details). Numbers of micrographs obtained (fourth rows), selected for scanning (third rows), processed by MRC program (second rows) and finally merged into a 3D structure (first rows) are indicated. Because all of the tilted images were affected by the image-shift due to specimen charging in the case of the single carbon preparation of the *E2*AlF crystals, micrographs with recognizable diffraction spots in the direction parallel to the tilt axis (as shown in Fig. 4d) were selected (*a*).

## Results and discussion

### ‘Hydrophilic’ 2D crystals from gastric H^+^,K^+^-ATPase

Gastric H^+^,K^+^-ATPase is an ATP-driven proton pump responsible for gastric acid secretion [25] that comprises a catalytic α-subunit (∼100 kDa) and an accessary β-subunit (∼35 kDa). The α-subunit contains 10 transmembrane helices in which cation-binding sites are located, and large cytoplasmic domains (A, P, N domains) where ATP-hydrolysis occurs; the large cytoplasmic domains comprise ∼70% of the total mass of the α-subunit [26]. The β-subunit has a single transmembrane helix with a short (∼30 amino acids) N-terminal cytoplasmic tail and a C-terminal ectodomain. Like other P-type ATPases, vectorial cation transport is accomplished by cyclical conformational changes between two principal functional states (*E1* and *E2*) and their corresponding phosphoenzyme intermediates (*E1*P and *E2*P) [27].

**Fig. 1.**
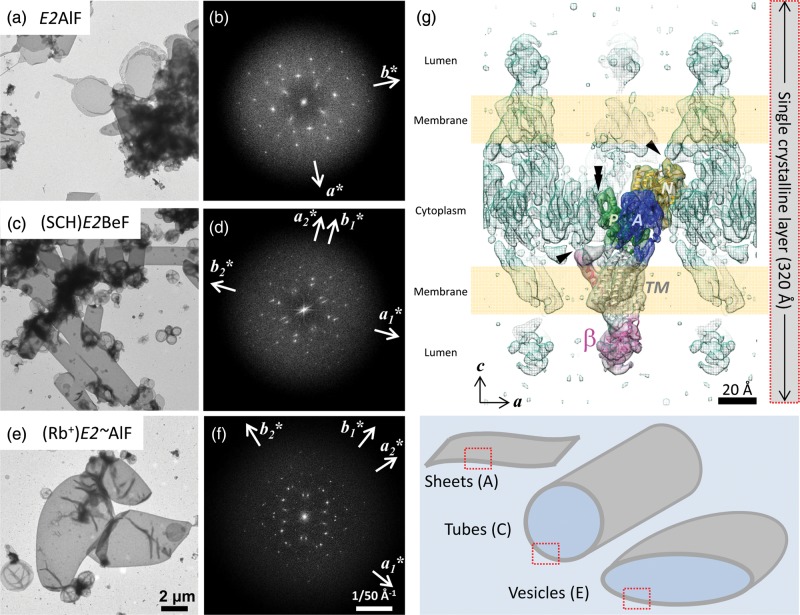
Negatively stained 2D crystals of H^+^,K^+^-ATPase at different conformations show the variety in their morphologies. Flat-sheet crystals of *E2*AlF (a) consist of a single-crystalline array, shown as a single reciprocal lattice in its Fourier transformation (b, indicated as a* and b*). On the other hand, flattened tubular crystals of (SCH)*E2*BeF (c) and vesicular crystals of (Rb^+^)*E2*AlF (e), and their Fourier transformations (d and f, respectively) show two overlapping reciprocal lattices (*a*_1_*, *b*_1_* and *a*_2_*, *b*_2_*). Thus, each layer of these crystals was processed independently as two overlapping crystalline layers. Scale bars for crystal images and their Fourier transforms are shown in panel E (2 μm) and panel F (1/50 Å^−1^), respectively. (g) Crystal packing of (SCH)*E2*BeF crystals shows that the inter-molecular contact between the N-terminal tail of β-subunit and the N domain (arrowhead), and the protruded structure of the P domain and the outermost portion of the A domain (double arrowhead) at the cytoplasmic side of the molecule. Color surface indicates EM map of H^+^,K^+^-ATPase αβ-protomer (blue, A domain; yellow, N domain; green, P domain; light gray, TM helices; pink, β-subunit) with a superimposed ribbon model. Green mesh indicates symmetry-related neighboring molecules and wheat-colored boxes indicate approximate locations of lipid bilayers. Due to crystal packing, the single-crystalline layer (indicated by a grey bar) consists of two lipid bilayers (indicated as wheat-colored boxes), which is responsible for the one-crystalline layer of each crystal in the different morphologies (red dotted boxes in lower panel cartoon). For interpretation of colour in this figure, the reader is referred to the web version of this article.

Successful 2D crystallization of gastric H^+^,K^+^-ATPase has yielded 2D crystals with different morphologies, including sheets (Fig. 1a and b) [19], tubes (Fig. 1c and d) [18,20,28] and vesicles (Fig. 1e and f) [29], depending on the conformational state of the protein and other crystallization conditions, although their crystal packing is essentially the same (Fig. 1g). Single-crystalline sheets consist of two-membrane layers, and the proteins in the two-membrane layers are related to each other by a two-fold screw axis, resulting in a *p22_1_2_1_* symmetry. Thus, the crystal ideally forms planar crystalline arrays like the single-layered sheet crystals found in the *E2*AlF conformation (Fig. 1a and b). For tubular crystals (Fig. 1c and e), however, they are too thick to apply a helical symmetry like the thin tubular crystals of acetylcholine receptors [30]. Thus, as is the case for vesicular crystals, tubular crystals are analyzed as two overlapping layers of crystalline sheets (Fig. 1d and f). Owing to its characteristic crystal packing, all of the inter-molecular contacts in the H^+^,K^+^-ATPase 2D crystals can be found at the cytoplasmic portions of the molecules (Fig. 1g), in marked contrast to relatively hydrophobic membrane proteins such as bacteriorhodopsin or aquaporins. Therefore, 2D crystals of H^+^,K^+^-ATPase are expected to be more susceptible to dehydration than those hydrophobic proteins.

### Carbon sandwich preparation effectively preserves crystal quality

**Fig. 2.**
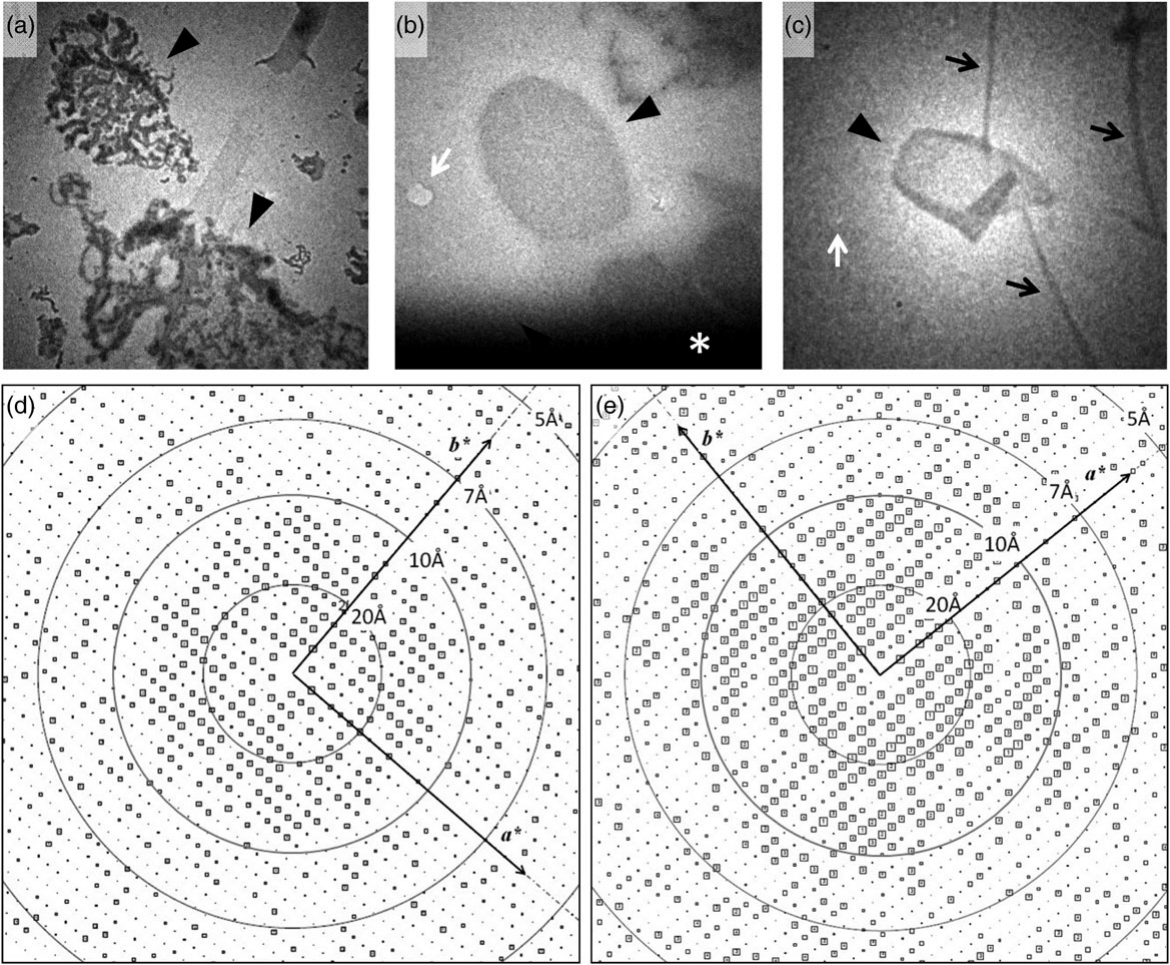
Comparison of the analyzed image quality between carbon sandwich and single carbon support film preparations. (a–c) Low magnification images (search mode) of H^+^,K^+^-ATPase 2D crystals (arrowheads) in frozen specimens prepared by the single carbon method (a and b) or carbon sandwich method (c). Most of the crystals embedded in the thin ice are dehydrated in the single carbon preparation (a), while preserved crystals can be found in the thicker ice area at the edge of the grid well (*: dark area) in some cases (b). In the carbon sandwich preparation, preserved 2D crystals are distributed evenly over the grid well (c). White arrows indicate the position used for focusing the image, and black arrows indicate crinkling of the carbon membrane, which usually occurs in carbon sandwich preparations. Because all images herein were obtained using a low-dose defocused diffraction mode, the mean diameter of ∼2 μm for sheet crystals is used for approximate scaling. (d and e) IQ-plot calculated from a non-tilted image of H^+^,K^+^-ATPase single sheet crystal in the *E2*AlF conformation prepared by the single carbon support film (d) and carbon sandwich (e) techniques.

Trehalose-embedding has been applied for the successful structural analysis of several membrane proteins by preserving 2D crystals in the hydrated state [11]. When we applied standard single carbon support film preparations for apparently flat sheet crystals made of decylmaltoside-solubilized H^+^,K^+^-ATPase in the presence of AlF adopting a *E2*AlF conformation (Fig. 1a), most of the crystals were obviously broken (Fig. 2a) and poorly ordered, as evaluated in the optical diffraction. Exceptions were observed at the edge of the grid well (indicated by the asterisk in Fig. 2b), in which remaining embedding buffer provides relatively thicker vitrified ice than that in the center of the grid well (Fig. 2a). In such a wet environment, some of the crystals were preserved and diffraction spots reach ∼10 Å resolution (Fig. 2d) in its calculated Fourier components (IQ-plot) [6].

**Fig. 3.**
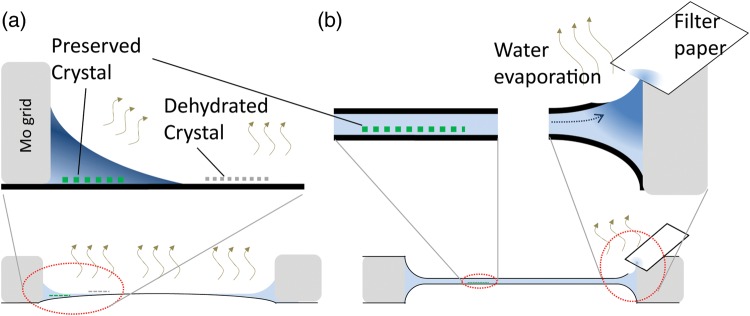
Cartoons depicting the cross section of the specimen prepared by single carbon support film (a) and that by carbon sandwich technique (b). Black lines indicate carbon support films, gray boxes indicate the molybdenum grid and light blue indicates the embedding buffer. As water evaporated (tan wavy arrows) from the surface of the specimen (a), most of the crystals were broken by dehydration (grey dotted line). Preserved crystals (green dotted line) were always observed in the thick ice at the edge of the grid (Fig. 2b), while the resolution is limited (Fig. 2d) due to the increased concentration of the embedding buffer by dehydration (dark blue). In contrast, water evaporation occurred only at the blotting position where the upper carbon membrane was partially broken by filter paper in the carbon-sandwiched specimen (b). Thus, the concentration change of the embedding buffer was limited around 2D crystals sandwiched between two carbon films (Fig. 2c), while the salt concentration was increased at the blotting position (b). Removal of excess water by blotting or evaporation induced spontaneous water flow (dotted arrow), which may prevent free diffusion of concentrated reagents to the central area of the grid. Therefore, the microenvironment of the 2D crystals embedded in the thin water layer seems to remain constant during preparation, and thus the inherent crystal qualities are preserved in the carbon sandwich preparations (Fig. 2e). For interpretation of colour in this figure, the reader is referred to the web version of this article.

Using the same batch of 2D sheet crystals of *E2*AlF H^+^,K^+^-ATPase, the qualities of non-tilted images prepared by the carbon sandwich technique (Fig. 2c) were compared with those supported by a single carbon film. Non-tilted images from the carbon sandwich preparation can reach up to 7 Å of resolution (Fig. 2e), while those from single carbon preparations are limited to a resolution of 10 Å (Fig. 2d). The effect of the beam-induced image shift is excluded in this case because an isotropic Thon ring is clearly visible in the calculated Fourier transformation of both preparations under non-tilted conditions. Therefore, the remarkable difference in resolution is due to preservation of the 2D crystal quality during specimen preparation on the EM grid (Fig. 3). In carbon sandwich preparations, the crystals are sandwiched between two carbon films and are thus likely protected from rapid dehydration (Fig. 3b), while in single carbon preparations, the upper surface is in direct contact with the air atmosphere, and water is easily evaporated causing excess dehydration as well as associated changes in the embedding buffer such as variable salt concentrations (Fig. 3a).

The H^+^,K^+^-ATPase molecules are interconnected at their hydrophilic part to form crystals in the 2D crystallization process (Fig. 1g). Thus, excess dehydration and/or concentration changes of the reagents in the embedding buffer may destroy both the inter- and intra-molecular interactions, leading to deterioration of the inherent crystal quality and even the original protein structure itself. In fact, a change in pH and/or salt concentration in the embedding buffer caused by washing 2D crystals on the grid induced disorder of the crystals within several seconds, as determined by negatively stained samples (data not shown). In the carbon sandwich preparations, the water layer on the EM-grid becomes thinner for a longer time by continuous blotting and spontaneous evaporation at the edge of the covered piece of carbon film (Fig. 3b). Because such events may induce spontaneous water flow toward the blotting position (indicated by the dotted arrow in Fig. 3b), free diffusion of the concentrated reagent from this position should be suppressed. Therefore, while salt concentration is increased around the blotting position (indicated as the dark blue region at the edge of the specimen in Fig. 3b), the salt concentration of the solution in most of the other areas, which contain many crystals (light blue region in Fig. 3b), is presumed to be less variable. Compared with the single carbon preparation, the carbon sandwich preparation protects the 2D crystals from environmental changes such as dehydration as well as salt concentration variability, and thus structural information can be extracted with its full potential for electron crystallography.

### Compensation effect of carbon sandwich preparation against beam-induced image shift

**Fig. 4.**
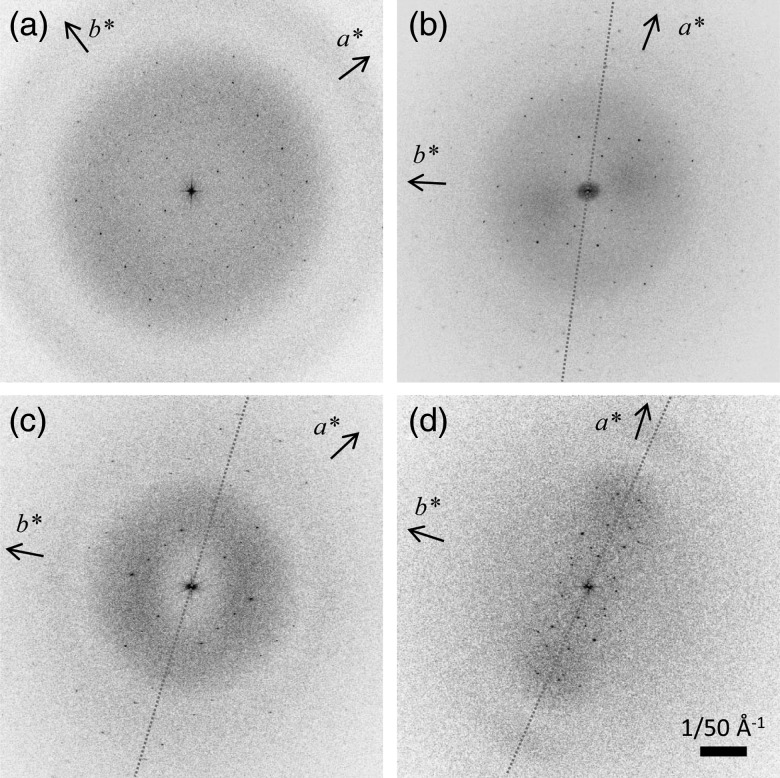
Representatives of Fourier transforms of a crystal image taken from non-tilted (a), 45° (b) and 70° (c) tilted specimens prepared by the carbon sandwich technique, show isotropic diffraction spots as well as Thon rings and are thus well suited for image processing [19]. In contrast, spots just along the tilt axis (dotted lines) are visible in the Fourier transform of 20° tilted crystals from single carbon preparation due to beam-induced specimen charging (d). Reciprocal lattice vectors (a*, b*) are indicated as arrows, and tilted axes are shown as dotted lines in tilted data. A scale bar is shown in panel D (1/50 Å^−1^).

Image shift due to beam-induced specimen charging causes diffraction spots perpendicular to the tilted axis to disappear in the Fourier transforms of images, even at medium or low resolution, as shown in Fig. 4d. The efficiency of isotropic data collection without deterioration of images of the tilted specimen is, therefore, another key requirement for determination of the 3D structure with a reduced missing cone effect. In the case of H^+^,K^+^-ATPase 2D crystals, the image shift severely affects the crystal images obtained from single carbon preparations, presumably because this molecule forms thicker 2D crystals, which accumulate more charge than thinner ones. It is notable that more than 20% of micrographs are affected by image shift, even those of non-tilted specimens, and no micrographs without an image shift have ever been obtained from tilted specimens prepared by a single carbon support film (Table 1). In contrast, although the success ratio for collecting images of bacteriorhodopsin 2D crystals was not very high, the quality of ∼2% of the images was high, even those obtained at a 60° tilt. Because of the hydrophilic molecular packing in H^+^,K^+^-ATPase crystals, preserved crystals are always embedded in relatively thick vitrified ice, which can usually be found at the edge of the molybdenium grid well in single carbon preparations (Figs. 2b and 3a), such a thick specimen area more easily accumulates charges that deflect the electron beam and cause an image shift [17]. More importantly, ice thickness, which is steeply variable at the edge of the specimen as seen in Figs. 2b and 3a, accumulates charge unevenly, resulting in a more serious deflection of the beam. This is another reason why more than 20% of micrographs of H^+^,K^+^-ATPase crystals are affected by an image shift even in non-tilted specimen conditions, while all images of bacteriorhodopsin crystals in the non-tilted condition provided isotropic data in their Fourier transforms.

Consistent with a previous study of aquaporin-4 crystals [17], the carbon sandwich preparation dramatically improved the efficiency of data collection even from samples susceptible to beam-induced image shift (Fig. 4). While the success rate depended on the specimen thickness as well as the surrounding conditions of the specimen, including the objective-lens aperture, fewer than 10% of images were affected by beam-induced image shift for a 20° tilted specimen and fewer than 30–40% of images were affected by a beam-induced image shift for a greater than 45° tilted specimen (Fig. 4a–c). Isotropic data can be successfully collected even from 70° tilted carbon-sandwiched specimens (Fig. 4c), while, even at 20° tilted specimen, diffraction spots and a Thon ring perpendicular to the tilt axis disappeared in the Fourier transform of the crystal image produced by a single carbon preparation (Fig. 4d).

### Efficiency of data collection achieved with the carbon sandwich technique

Statistics of the data collection for the structural analysis of H^+^,K^+^-ATPase in different conformations as well as by two specimen preparation techniques in the *E2*AlF state are summarized in Table 1. Qualities of all of the micrographs obtained (fourth rows) were assessed by optical diffraction, and selected images were digitized (third rows). In this step, except for single carbon prepared samples, micrographs with poorly ordered crystals and/or beam-induced image shift were excluded (58% of total micrographs). Selected micrographs (42% of total micrographs) were then processed using the MRC image processing program and selected manually based on the amplitudes of IQ plots (second rows). The acceptable images (27% of total micrographs) were further selected by phase residual cut-off, and finally ∼15% of the total micrographs obtained were merged into the 3D structure factor (first rows). These results markedly contrast with the statistics of the single carbon preparation, by which no structure could be analyzed regardless of how many images were obtained (1213 images).

**Fig. 5.**
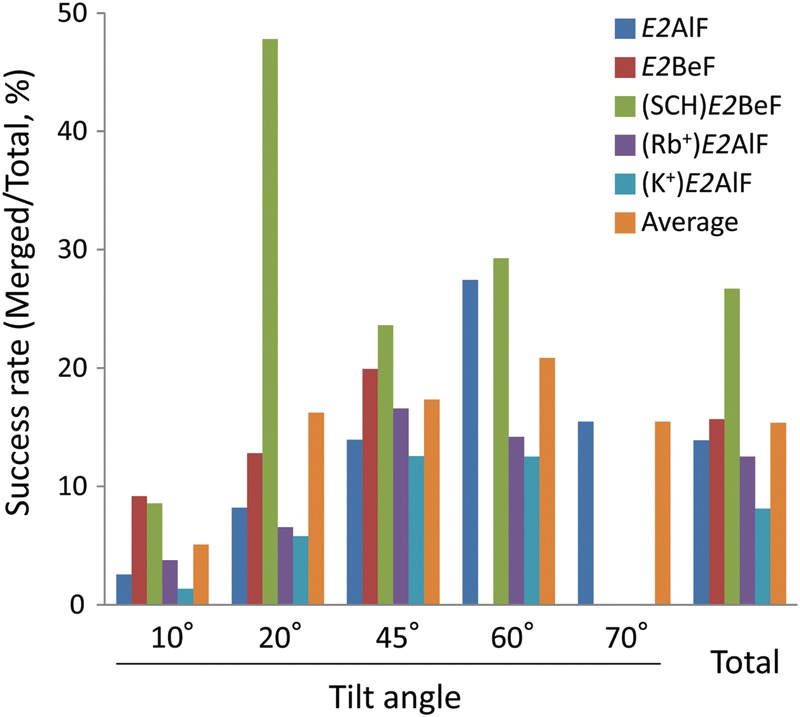
Statistics of data collection from carbon-sandwiched specimens. Success rate was calculated from the number of micrographs merged into the 3D structure factor per total number of micrographs obtained, for each crystal sample in a different conformation of the transport cycle as indicated by different colors in the figure. For interpretation of colour in this figure, the reader is referred to the web version of this article.

Statistics of the success rate for merging (i.e. number of micrographs merged/total) are summarized in Fig. 5. To obtain a reliable structure, analyzed data were carefully selected according to their phase residuals and amplitude IQ values, as indicated above. Because strict criteria for the selection were set, especially for 0° and 20° tilted data, the success rate in these lower tilt angles was limited, except for a 20° tilt of (SCH)*E2*BeF, compared with those having high-tilt angles. There was an exceptionally high success rate in the 20° tilted (SCH)*E2*BeF crystal (48% of total micrographs obtained were merged into the 3D structure factor), in marked contrast to the success rate of other 20° tilted data (6–16%). The reason for this is that all of the micrographs were obtained from only two successfully prepared grids. As seen in this example, and also the inconsistent success rates among other preparations, there was a large variety in the degree of preservation among carbon sandwich-prepared specimens. On the other hand, the (K^+^)*E2*AlF crystal had the lowest success rate among all the crystal samples (8% in average of all tilted angles), which cannot be explained by the variation in specimen preparation alone. Thus, the low efficiency in the (K^+^)*E2*AlF statistics is most likely due to the inherent quality of the crystals as well as their stability during specimen preparation. For H^+^,K^+^-ATPase samples, successfully or poorly preserved crystals are hardly distinguishable based on their morphology alone in the low-dose search mode, and data collection still relies on trial-and-error due to the large variation in crystal preservation. The average success rate of 15% is the mean of the values of 30 and 0%, rather than an even distribution of ∼15% preserved crystals. This indicates that further improvement in the reproducibility of crystal preservation is needed for efficient data collection.

## Concluding remarks

In this study, we show that the carbon sandwich preparation technique contributes to preservation of the inherent crystal quality. Preservation of the hydrated environment of 2D crystals made of membrane proteins with large hydrophilic domains achieved by the carbon sandwich preparation dramatically improves the resolution compared with single carbon support film preparation. We also provide updated statistics for the data collection required to determine previously published structures that were based on samples extremely susceptible to beam-induced image shift. Further improvement in the carbon sandwich preparation with highly reproducible preservation of 2D crystals is desired for more efficient data collection, which is indispensable for high-resolution structural analysis of fragile membrane proteins by electron crystallography.

## Funding

This research was supported by Grants-in-Aid for Scientific Research (S), National Institute of Biomedical Innovation, Japan New Energy and Industrial Technology Development Organization (NEDO), to Y.F., Grants-in-Aid for Young Scientists (B) to K.T., and Grants-in-Aid for Young Scientist (Start-up) and Platform for Drug Design, Discovery and Development from MEXT, Japan, to K. A. F.Y. was supported by a scholarship from MEXT, Japan. Funding to pay the Open Access publication charges for this article was provided by Grants-in-Aid for Scientific Research (S) to Yoshinori Fujiyoshi.

## References

[1] Henderson R, Unwin P N T (1975). Three-dimensional model of purple membrane obtained by electron
microscopy. Nature.

[2] Fujiyoshi Y (1998). The structural study of membrane proteins by electron
crystallography. Adv. Biophys..

[3] Henderson R, Baldwin J M, Ceska T A, Zemlin F, Beckmann E, Downing K H (1990). Model for the structure of bacteriorhodopsin based on high-resolution
electron cryo-microscopy. J. Mol. Biol..

[4] Glaeser R M, Downing K, DeRosier D, Chiu W, Frank J (2007). Electron crystallography of biological macromolecules.

[5] Amos L A, Henderson R, Unwin N (1982). Three-dimensional structure determination by electron microscopy of
two-dimensional crystals. Prog. Biophys. Mol. Biol..

[6] Henderson R, Baldwin J, Downing K, Lepault J, Zemiln F (1986). Structure of purple membrane from *Halobacterium halobium*:
recording, measurement and evaluation of electron micrographs at 3.5 Å
resolution. Ultramicroscopy.

[7] Henderson R, McMullan G (2013). Problem in obtaining perfect images by single-particle electron
cryomicroscopy of biological structures in amorphous ice. Microscopy (Tokyo).

[8] Fujiyoshi Y (2011). Electron crystallography for structural and functional studies of membrane
proteins. J. Electron Microsc. (Tokyo).

[9] Dubochet J, Chang J J, Freeman R, Lepault J, McDowall A W (1982). Frozen aqueous suspensions. Ultramicroscopy.

[10] Hirai T, Murata K, Mitsuoka K, Kimura Y, Fujiyoshi Y (1999). Trehalose embedding technique for high-resolution electron crystallography:
application to structural study on bacteriorhodopsin. J. Electron Microsc. (Tokyo).

[11] Chiu P-L, Kelly D F, Walz T (2011). The use of trehalose in the preparation of specimen for molecular electron
microscopy. Micron.

[12] Kimura Y, Vassylyev D G, Miyazawa A, Kidera A, Matsushima M, Mitsuoka K, Murata K, Hirai T, Fujiyoshi Y (1997). Surface of bacteriorhodopsin revealed by high-resolution electron
crystallography. Nature.

[13] Murata K, Mitsuoka K, Hirai T, Walz T, Agre P, Heymann J B, Engel A, Fujiyoshi Y (2000). Structural determinants of water permeation through
aquaporin-1. Nature.

[14] Hiroaki Y, Tani K, Kamegawa A, Gyobu N, Nishikawa K, Suzuki H, Walz T, Sasaki S, Mitsuoka K, Kimura K, Mizoguchi A, Fujiyoshi Y (2006). Implications of the aquaporin-4 structure on array formation and cell
adhesion. J. Mol. Biol..

[15] Gonen T, Cheng Y, Sliz P, Hiroaki Y, Fujiyoshi Y, Harrison S C, Walz T (2005). Lipid-protein interaction in double-layered two-dimensional AQP0
crystals. Nature.

[16] Koning R I, Oostergetel G T, Brisson A (2003). Preparation of flat carbon support films. Ultramicroscopy.

[17] Gyobu N, Tani K, Hiroaki Y, Kamegawa A, Mitsuoka K, Fujiyoshi Y (2004). Improved specimen preparation for cryo-electron microscopy using a
symmetric carbon sandwich technique. J. Struct. Biol..

[18] Nishizawa T, Abe K, Tani K, Fujiyoshi Y (2008). Structural analysis of 2D crystals of gastric
H^+^,K^+^-ATPase in different states of the transport
cycle. J. Struct. Biol..

[19] Abe K, Tani K, Nishizawa T, Fujiyoshi Y (2009). Inter-subunit interaction of gastric
H^+^,K^+^-ATPase prevents reverse reaction of the transport
cycle. EMBO J..

[20] Abe K, Tani K, Fujiyoshi Y (2011). Conformational rearrangement of gastric
H^+^,K^+^-ATPase induced by an acid
suppressant. Nat. Commun..

[21] Fujiyoshi Y, Mizusaki T, Morikawa K, Yamagishi H, Aoki Y, Kihara H, Harada Y (1991). Development of a superfluid helium stage for high-resolution electron
microscopy. Ultramicroscopy.

[22] Crowther R A, Henderson R, Smith J M (1996). MRC image processing programs. J. Struct. Biol..

[23] Tani K, Sasabe H, Toyoshima C (1996). A set of computer programs for determining defocus and astigmatism in
electron images. Ultramicroscopy.

[24] Fernandez J J, Sanjurjo J R, Carazo J M (1997). A spectral estimation approach to contrast transfer function detection in
electron microscopy. Ultramicroscopy.

[25] Ganser A L, Forte J G (1973). K^+^-stimulated ATPase in purified microsomes of bullfrog
oxynic cells. Biochim. Biophys. Acta.

[26] Toyoshima C, Nakasako M, Nomura H, Ogawa H (2000). Crystal structure of the calcium pump of sarcoplasmic reticulum at 2.6
Å resolution. Nature.

[27] Rabon E C, Reuben M A (1990). The mechanism and structure of the gastric H,K-ATPase. Annu. Rev. Physiol..

[28] Abe K, Tani K, Fujiyoshi Y (2010). Structural and functional characterization of
H^+^,K^+^-ATPase with bound fluorinated phosphate
analogs. J. Struct. Biol..

[29] Abe K, Tani K, Friedrich T, Fujiyoshi Y (2012). Cryo-EM structure of gastric H^+^,K^+^-ATPase
with a single occupied cation-binding site. Proc. Natl Acad. Sci. USA.

[30] Miyazawa A, Fujiyoshi Y, Unwin N (2003). Structure and gating mechanism of the acetylcholine receptor
pore. Nature.

